# Supplementary material for: Analysis of the oral microbiome composition of healthy individuals and the in vitro antibacterial activity of platelet-rich fibrin from these individuals against oral pathogenic bacteria
Source: Front Microbiol. 2025 Oct 30;16:1691046. doi: 10.3389/fmicb.2025.1691046 (PMC12612686; doi:10.3389/fmicb.2025.1691046)

Supplementary Material

1 Supplementary material 1: ‘Patient survey’ and ‘Patient examination form (API)’

PATIENT SURVEY

1. GENDER ....

2. AGE ....

3. HEIGHT ....

4. WEIGHT ....

5. Have you been diagnosed or have you ever suffered from any of the following conditions?

□ Diabetes

□ Atherosclerosis

□ Kidney diseases

□ Hypertension

□ Liver failure

□ Periodontal diseases

□ Cancer/oncological treatment – if yes, when did the treatment conclude?
....

□ AIDS

□ Other diseases (please specify)
....

6. Do you have a tendency to experience excessive bleeding? YES/NO

7. Are you currently pregnant? YES/NO

8. Do you smoke cigarettes? YES/NO

9. Are you allergic to anything? If yes, please specify: YES/NO
....

10. Have you taken antibiotics in the past month? YES/NO

11. Do you regularly take anti-inflammatory medications? YES/NO

12. Are you currently taking anticoagulant medications? YES/NO

13. Do your gums bleed during daily tooth brushing? YES/NO

14. How many times a day do you brush your teeth?
....

15. What additional hygiene practices do you engage in?

Flossing: YES/NO

Interdental brushes: YES/NO

Antiseptic mouth rinses: YES/NO

16. How many hours ago was your last hygiene treatment?
....

PATIENT EXAMINATION FORM (API)

Number of teeth in the oral cavity: ....

Number of teeth with caries: ....

Number of teeth lost due to caries and its complications: ....

The state of oral hygiene is assessed according to the Approximal Plaque Index (API) – the percentage ratio of the number of interproximal tooth surfaces displaying biofilm to the total number of examined interproximal surfaces.

Number of interproximal surfaces with plaque in the first quadrant (buccal aspect): ....

Number of interproximal surfaces with plaque in the second quadrant (palatal aspect): ....

Total number of interproximal surfaces examined in the first and second quadrants: ....

API equals: ....

2 Supplementary material 2: Table S1. The studied blood morphology parameters, reference values for adult people and average score with standard deviation

Abbreviation	Parameter	Unit	Adult normal ranges (man)	Adult normal ranges (woman)	Average score/standard deviation
WBC	Total White Blood Cell Count	10^3/µl	4.00-10.00	4.00-10.00	6.08/1.34
RBC	Red Blood Cells	10^6/ µl	4.00-6.30	3.80-5.20	5.15/0.58
HGB	Hemoglobin	g/dl	14.00-18.00	12.00-16.00	15.32/1.87
HCT	Hematocrit	%	40-54	37-47	45.37/4.43
MCV	Mean Corpuscular Volume	fl	80.0-97.0	80.0-97.00	88.27/2.8
MCH	Mean Corpuscular Hemoglobin	pg	27.0-32.0	27.0-32.0	29.74/1.14
MCHC	Mean Corpuscular Hemoglobin Concentration	g/dl	32.0-36.0	32.0-36.0	33.68/1.07
PLT	Platelets	10^3/µl	150-400	150-400	256.06/43.48
RDW-SD	Red Cell Distribution Width – Standard Deviation	fl	39-46	39-46	40.2/2.83
RDW-CV	Red Cell Distribution Width – Coefficient of Variation	%	11.00-14.00	11.00-14.00	12.37/0.65
PDW	Platelet Distribution Width	fl	10.8-16.8	10.8-16.8	12.47/2.32
MPV	Mean Platelet Volume	fl	7.5 – 11.5	7.5 – 11.5	10.46/1.05
P-LCR	Platelet Large Cell Ratio	%	18.2-40.2	18.2-40.2	28.52/7.89
PCT	Plateletcrit	%	0.19-0.33	0.19-0.33	0.27/0.04
NRBC	Nucleated Red Blood Cells	10^3/µl	0.00-0.10	0.00-0.10	0
NEUT	Neutrophils	10^3/µl	1.50-7.50	1.50-7.50	3.5/1.15
LYMPH	Lymphocytes	10^3/µl	1.00-4.00	1.00-4.00	1.87/0.52
MONO	Monocytes	10^3/µl	0.10-1.30	0.10-1.30	0.52/0.1
EO (EOSYNO)	Eosinophils	10^3/µl	1.00-3.00	1.00-3.00	0.12/0.08
BASO	Basophils	10^3/µl	0.00-0.2	0.00-0.2	0.03/0.01
IG	Immature Granulocytes	10^3/µl	0.00-0.04	0.00-0.04	0.04/0.03
RET	Reticulocytes	10^6/µl	0.025 – 0.075	0.025 – 0.075	0.07/0.02
IRF	Immature Reticulocyte Fraction	%	1.6 – 12.1	1.6 – 12.1	4.93/2.24
LFR	Low Fluorescence Ratio	%	87.89 – 98.37	87.89 – 98.37	95.1/2.24
MFR	Medium Fluorescence Ratio	%	1.60–11.04	1.60–11.04	4.53/2.04
HFR	High Fluorescence Ratio	%	0.00–2.65	0.00–2.65	0.39/0.29
RET-He	Reticulocyte Hemoglobin Equivalent	pg	28.8–39.9	28.8–39.9	31.21/1.34
AS-LYMPH	Atypical Lymphocytes	10^3/µl	0.00-0.00	0.00-0.00	0.006/0.001
RE-LYMPH	Reactive Lymphocytes	10^3/µl	0.00-0.50	0.00-0.50	0.05/0.05
Micro R	Microcytic Red Cells	%	0.2-4.8	0.2-4.8	1.34/0.64
Macro R	Macrocytic Red Cells	%	3.3-4.8	3.3-4.8	3.87/0.5
Hypo He	Hypochromic Red Cells	%	0.1–0.7	0.1–0.7	0.16/0.11
FRC	Fragmented Red Cells	10^6/µl	0-0.2	0-0.2	0
NEUR-RI	Neutrophil Reactivity Index	FI	39.8-51.0	39.8-51.0	44.89/2.08
NEUT-GI	Neutrophil Granularity Index	ST	142.8-159.3	142.8-159.3	152.13/5.03

3 Supplementary material 3: Microbiome biodiversity indexes comparison between female and male patients

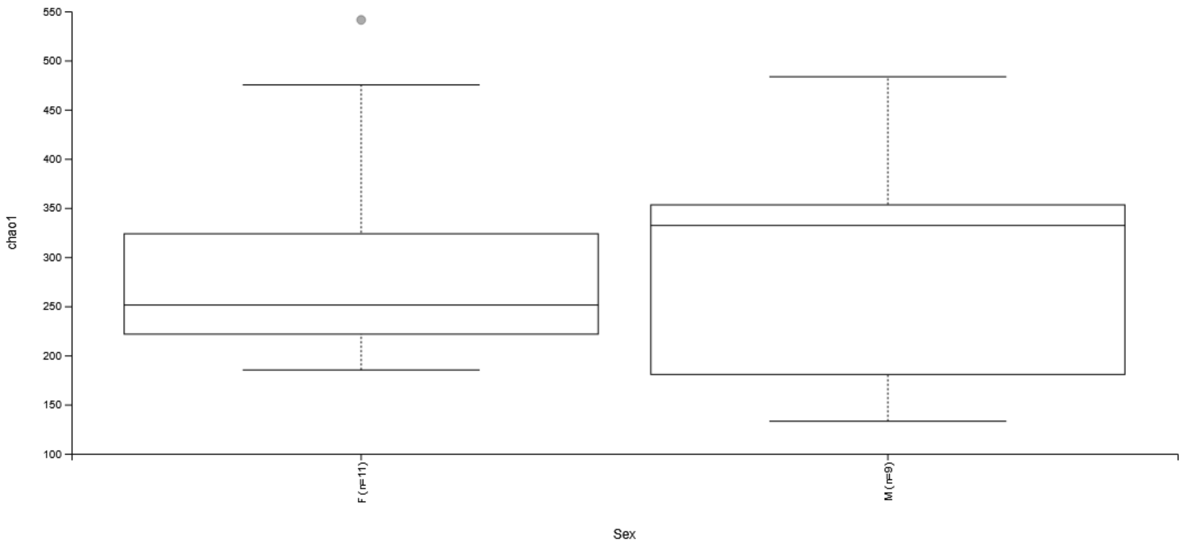

Figure S1. The boxplot with confidence intervals, showing the data distribution for Chao1 biodiversity index for 16S rRNA for samples divided by sex.

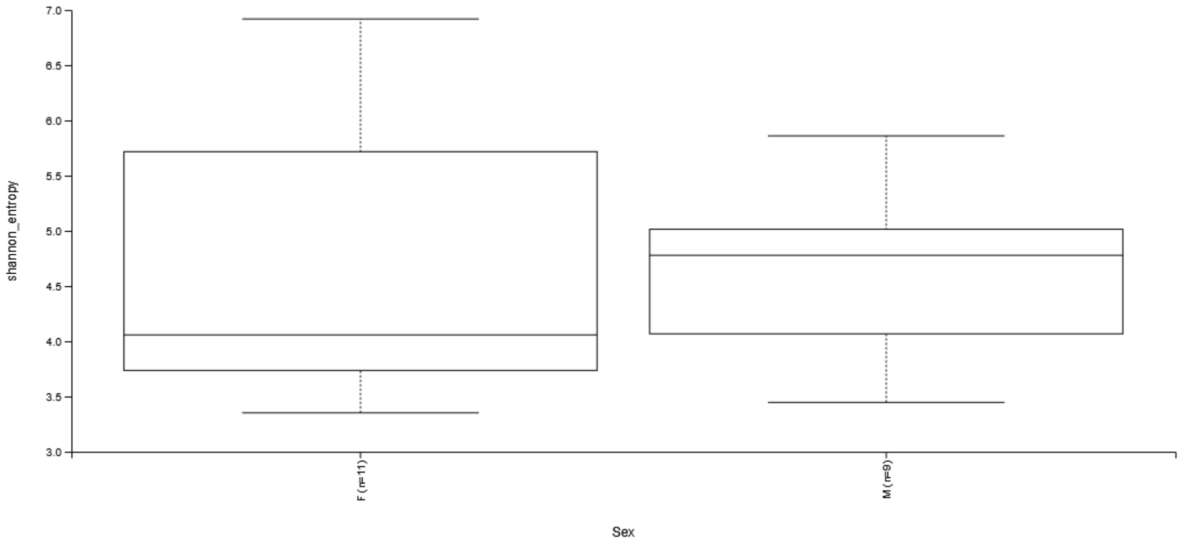

Figure S2. The boxplot with confidence intervals, showing the data distribution for Shannon biodiversity index for 16S rRNA for samples divided by sex.

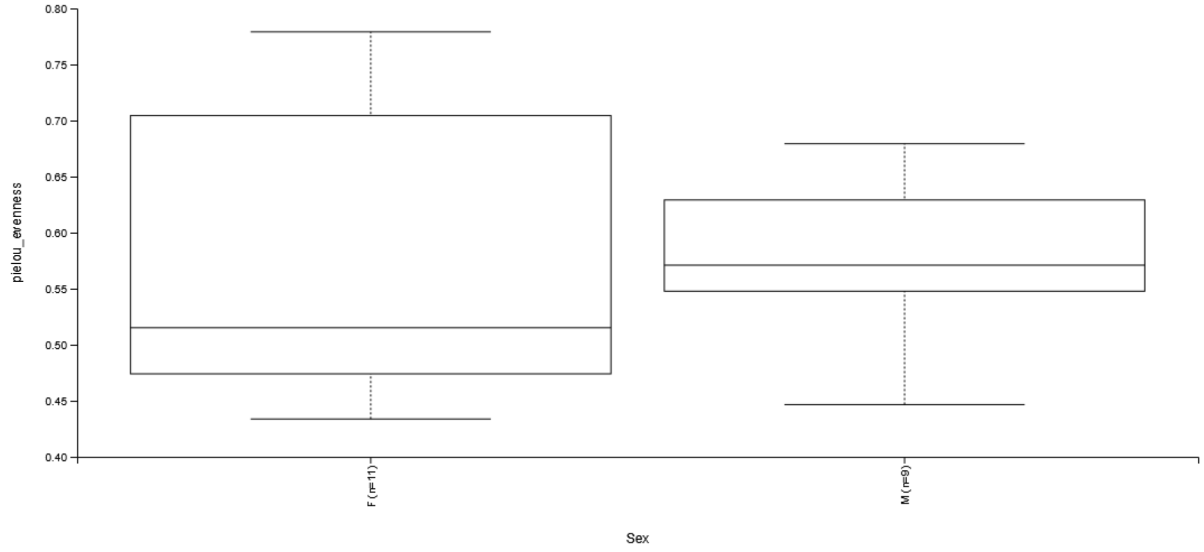

Figure S3. The boxplot with confidence intervals, showing the data distribution for Pielou biodiversity index for 16S rRNA for samples divided by sex.

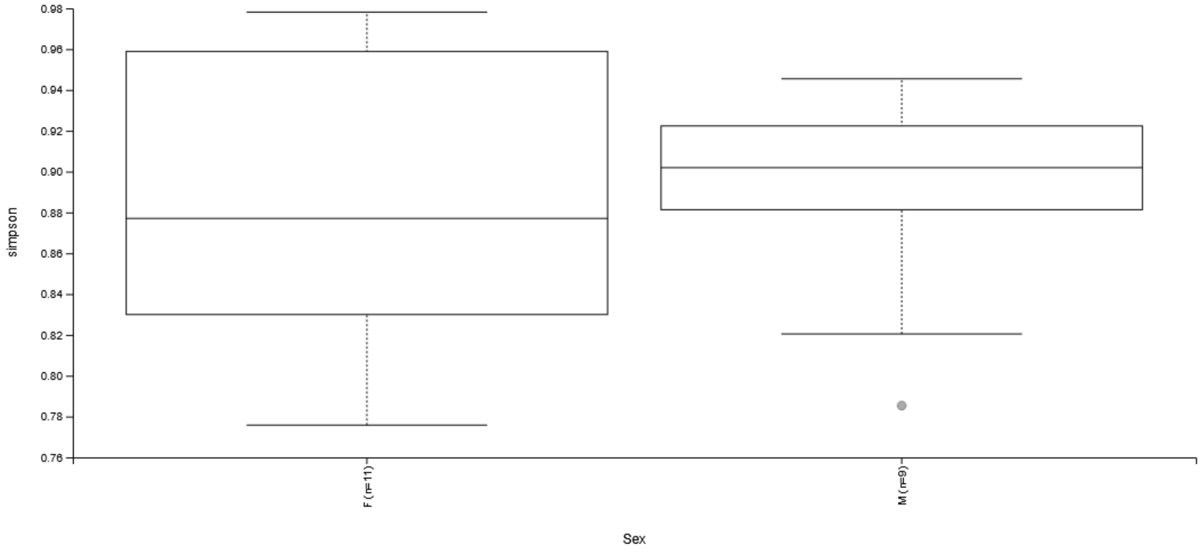

Figure S4. The boxplot with confidence intervals, showing the data distribution for Simpson biodiversity index for 16S rRNA for samples divided by sex.

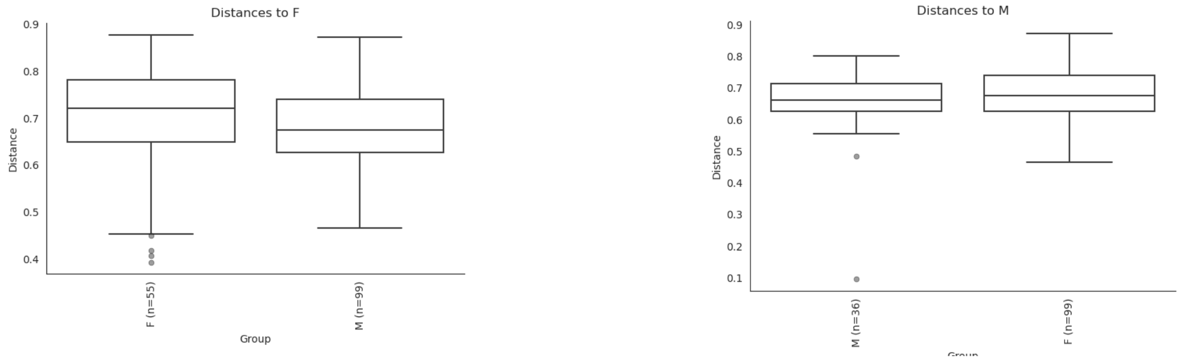

Figure S5. The boxplot with confidence intervals, showing the data distribution for Bray-Curtis index for 16S rRNA for samples divided by sex.

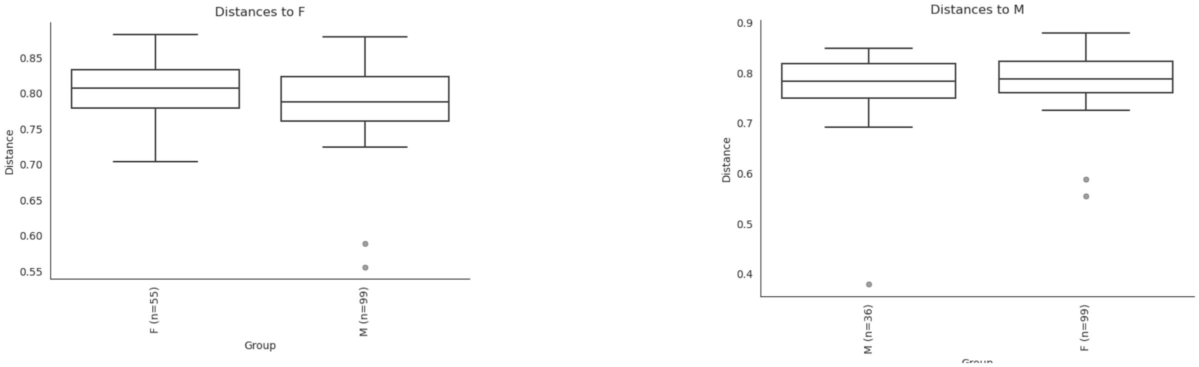

Figure S6. The boxplot with confidence intervals, showing the data distribution for Jaccard index for 16S rRNA for samples divided by sex.

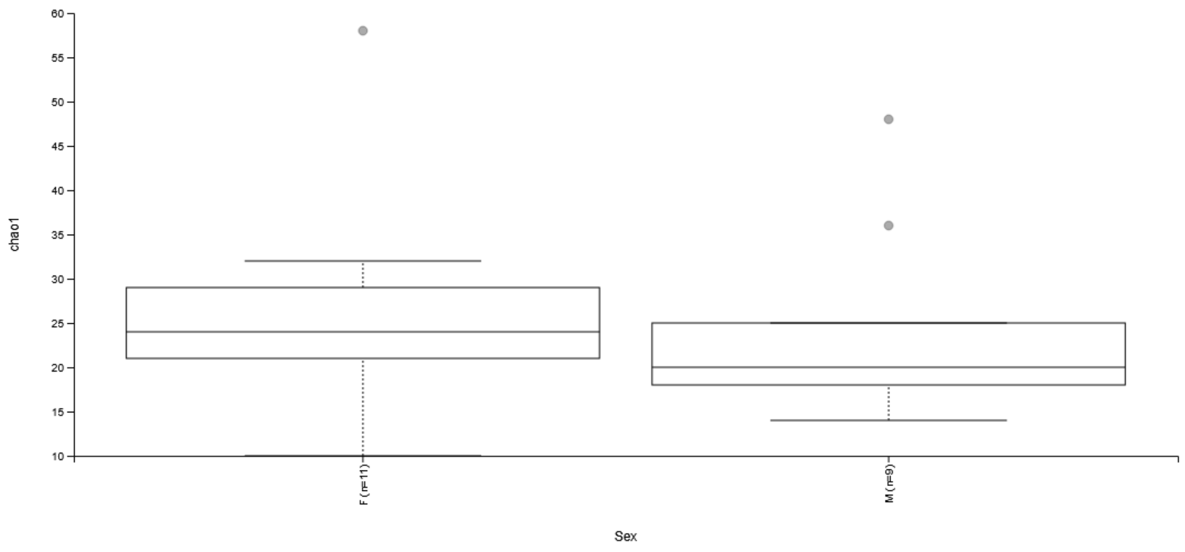

Figure S7. The boxplot with confidence intervals, showing the data distribution for Chao1 biodiversity index for ITS for samples divided by sex.

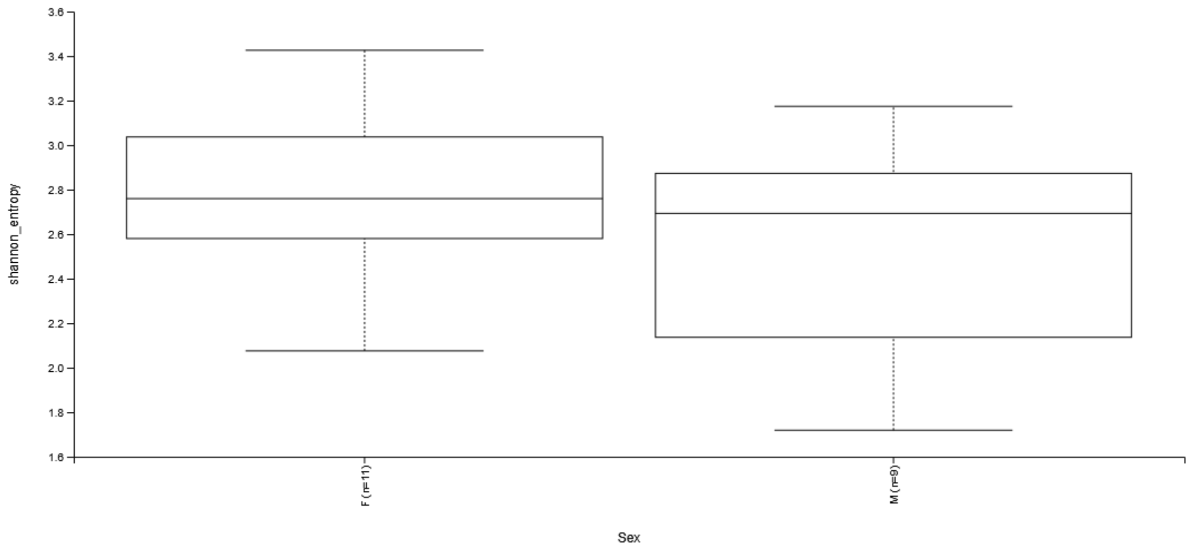

Figure S8. The boxplot with confidence intervals, showing the data distribution for Shannon biodiversity index for ITS for samples divided by sex.

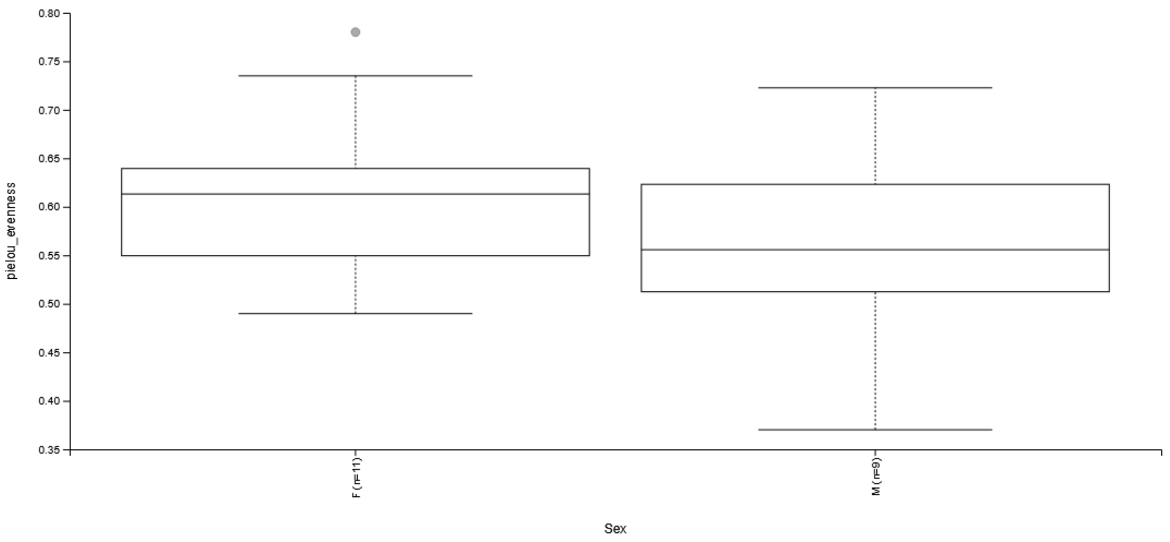

Figure S9. The boxplot with confidence intervals, showing the data distribution for Pielou biodiversity index for ITS for samples divided by sex.

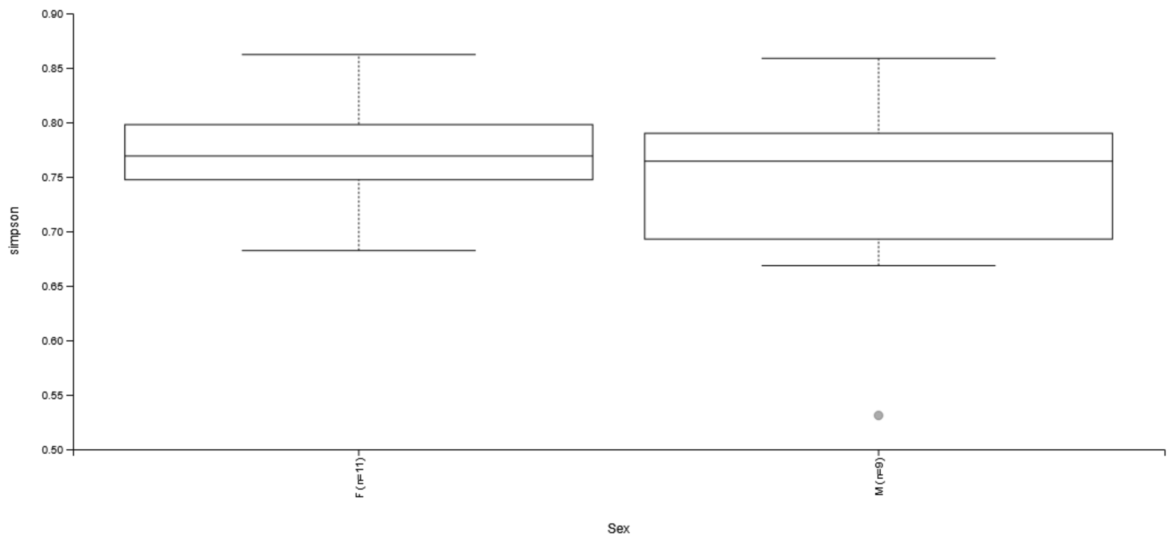

Figure S10. The boxplot with confidence intervals, showing the data distribution for Simpson biodiversity index for ITS for samples divided by sex.

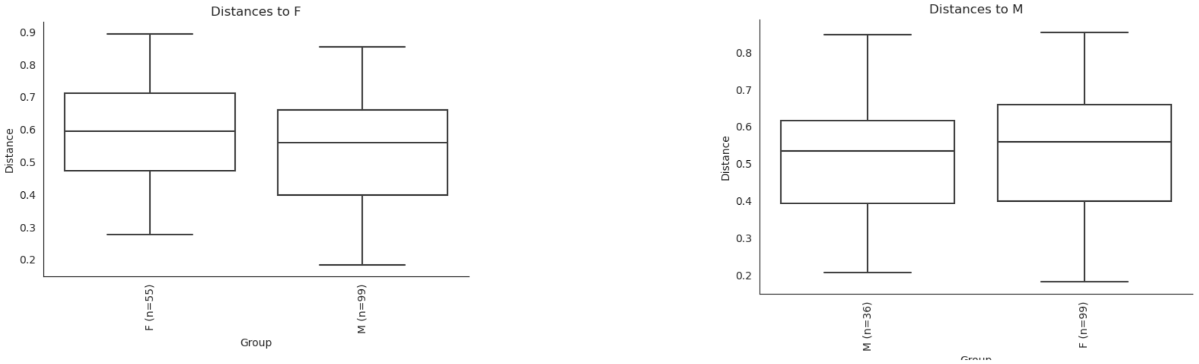

Figure S11. The boxplot with confidence intervals, showing the data distribution for Bray-Curtis index for ITS for samples divided by sex.

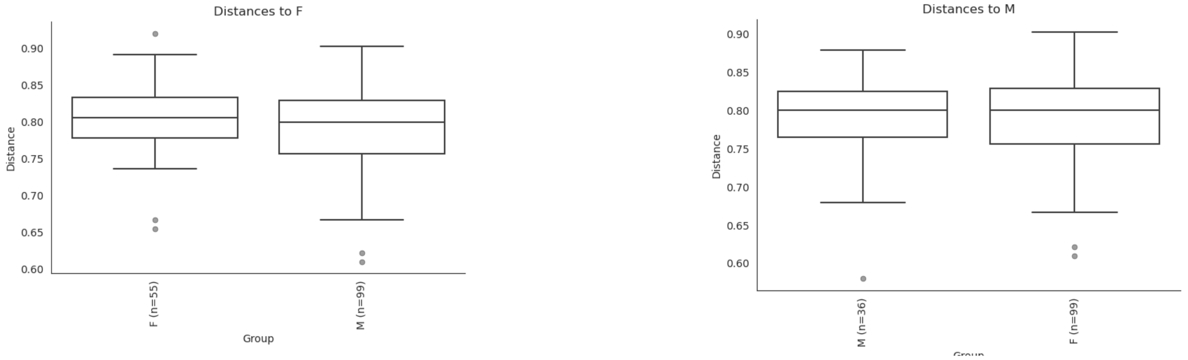

Figure S12. The boxplot with confidence intervals, showing the data distribution for Jaccard index for ITS for samples divided by sex.

4 Supplementary material 4: Photographic documentation - selected sample photos

A. The inhibition zone - antibacterial activities of the A-PRF membrane and LP fraction (according to the methodology section 2.6 and results section 3.4 in the manuscript)

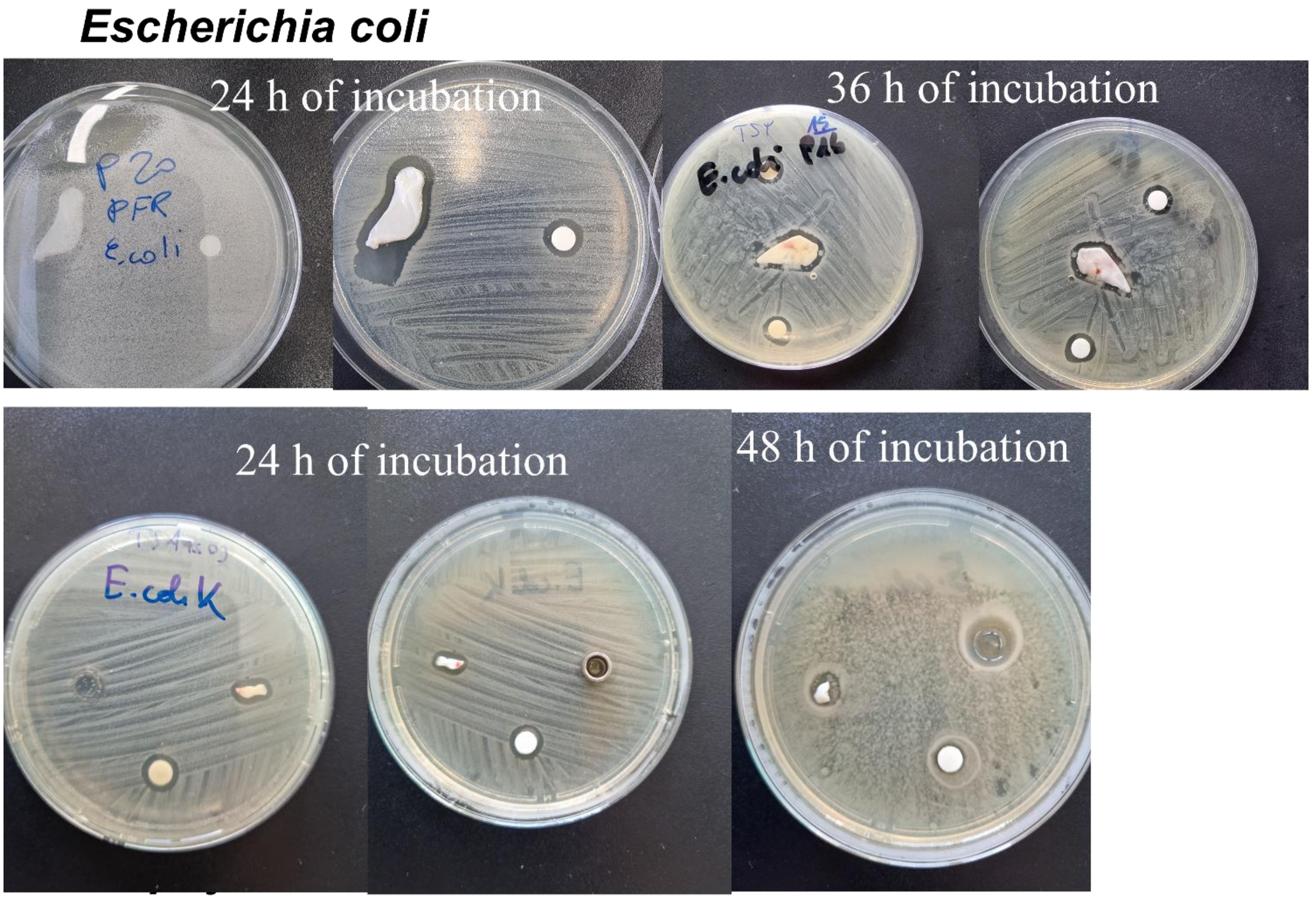

Enterococcus casseliflavus

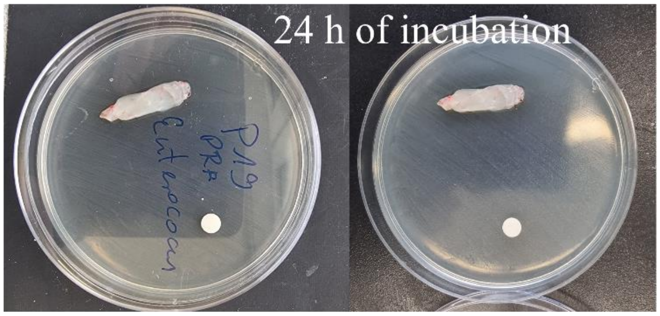

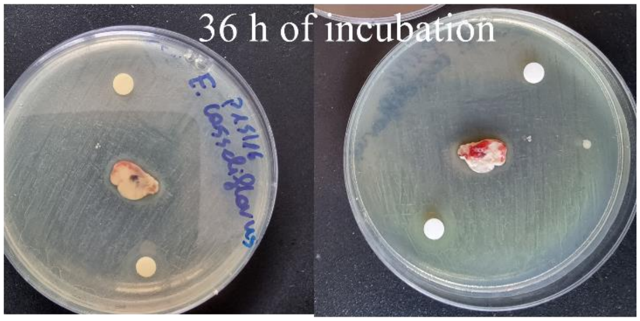

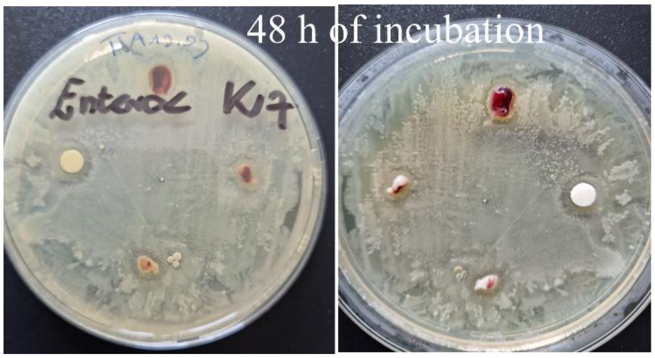

Bacillus subtilis

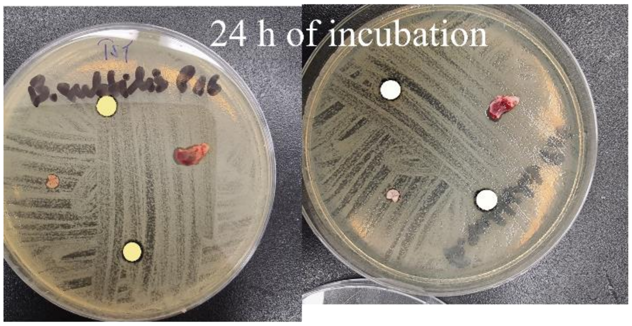

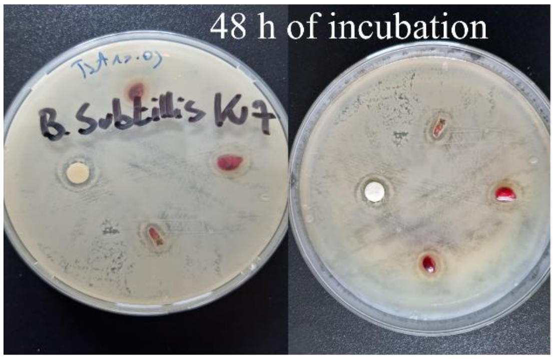

Micrococcus luteus

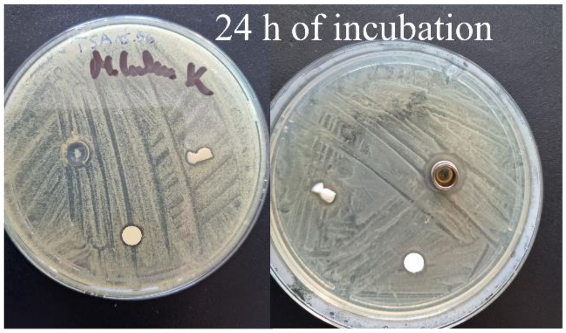

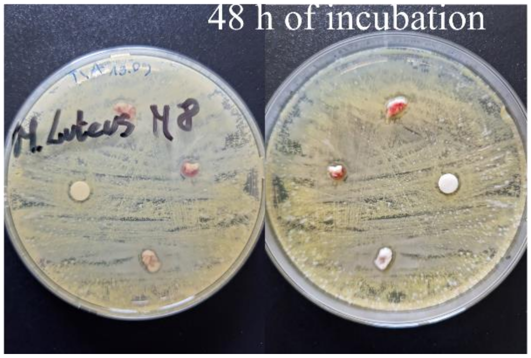

Staphylococcus lentus

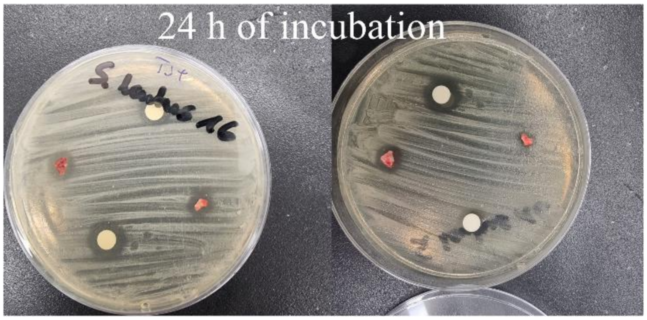

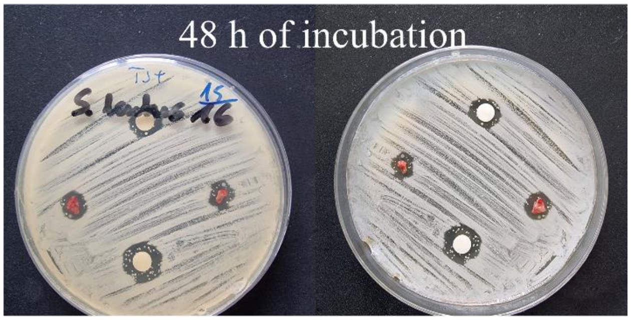

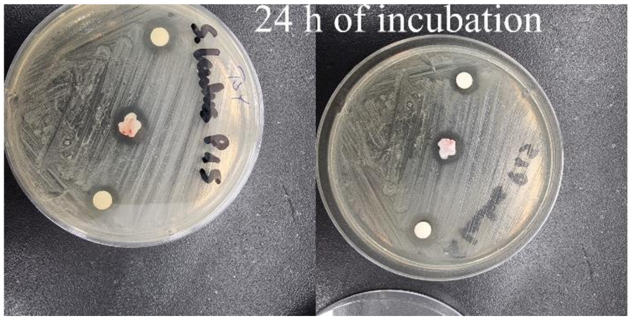

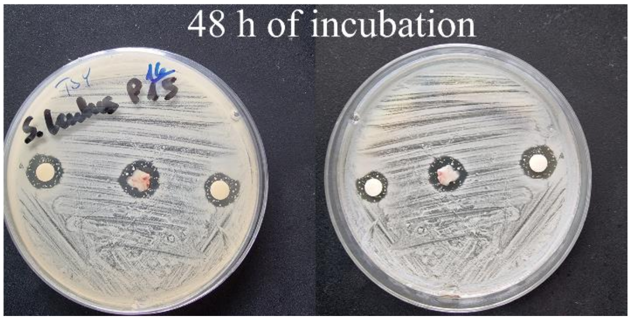

Streptococcus mutans

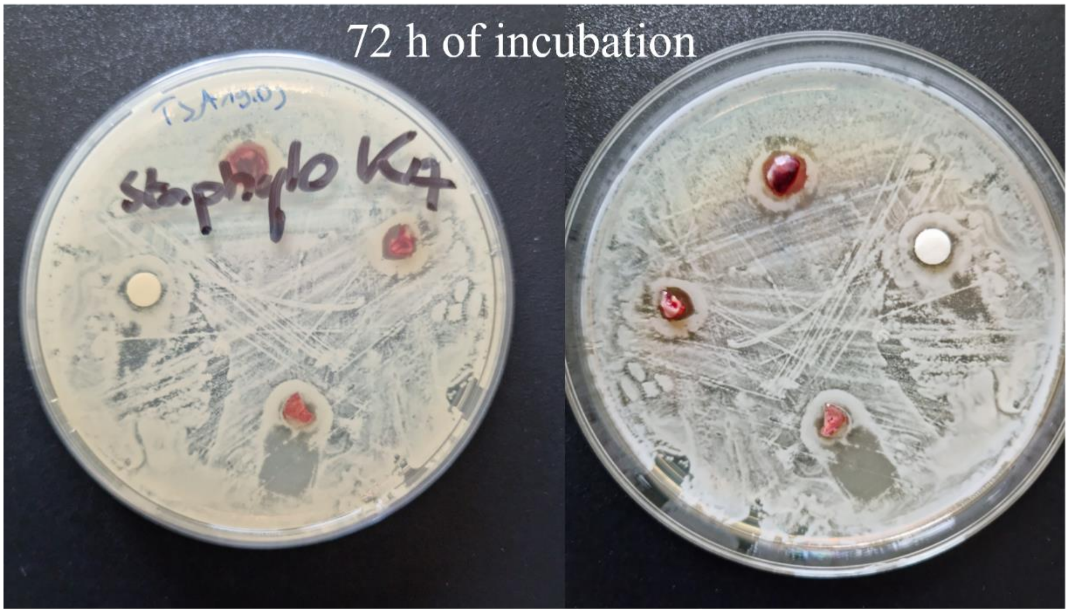

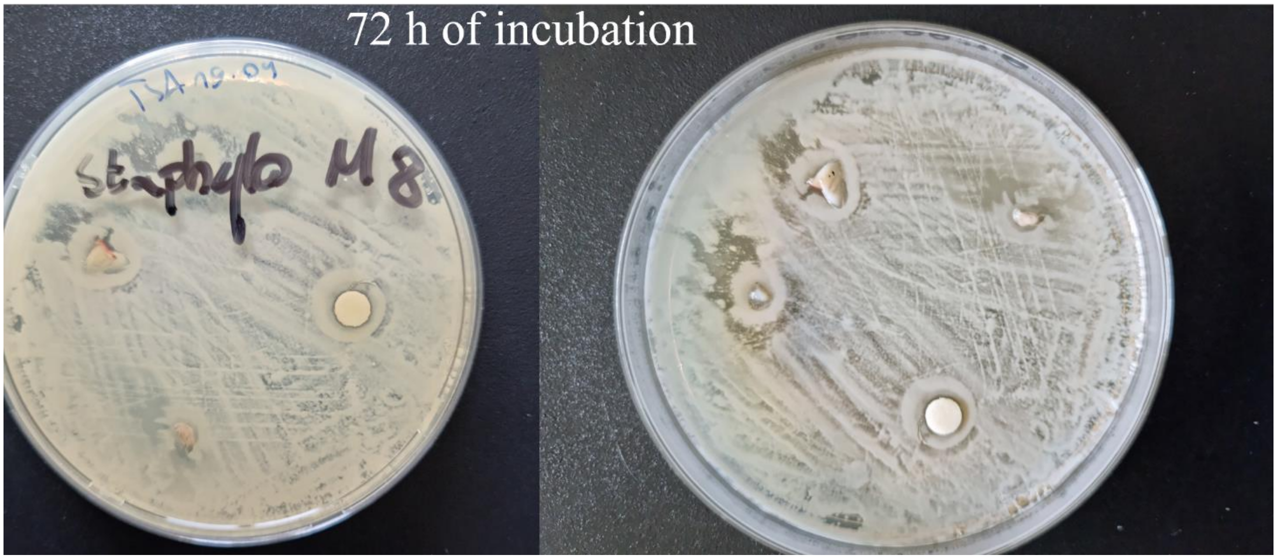

Porphyromonas gingivalis

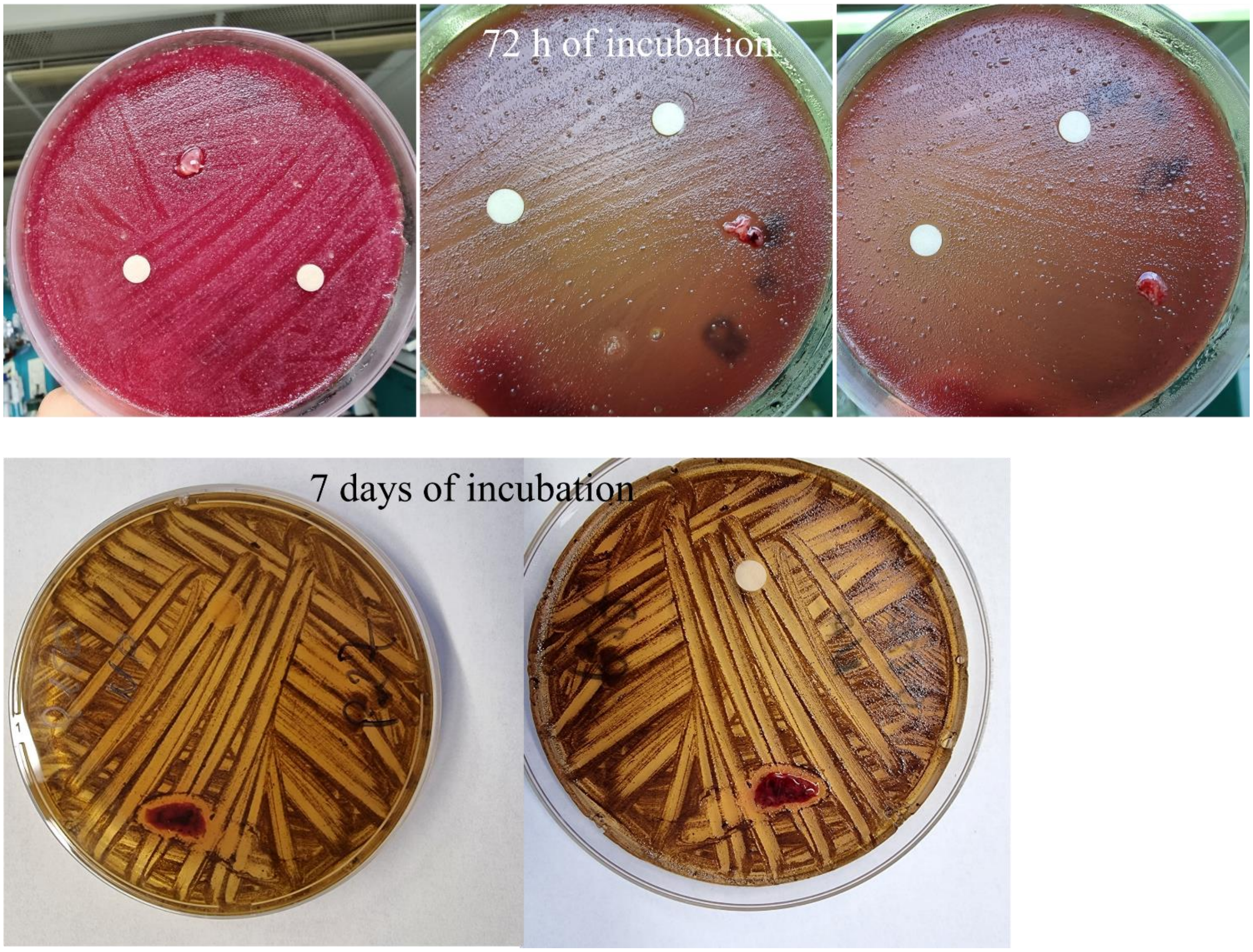

B. Antibacterial activities of whole A-PRF membrane against *E. coli* and no antibacterial activity against *P. gingivalis* (according to the methodology section 2.6 and results section 3.4 in the manuscript; K: positive control)

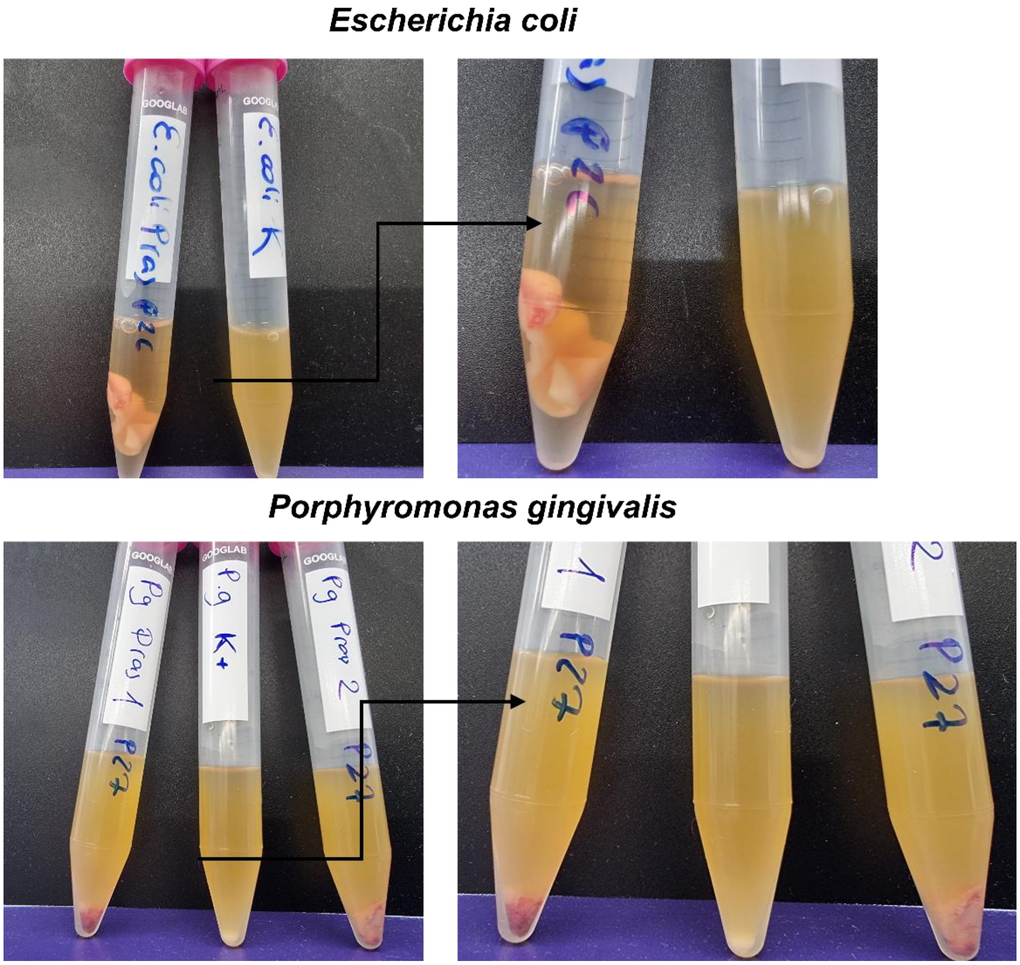

Supplement: Supplementary file 1 [file Data_Sheet_1.pdf]
